# Supplementary material for: MAPKs Are Highly Abundant but Do Not Contribute to α1-Adrenergic Contraction of Rat Saphenous Arteries in the Early Postnatal Period
Source: Int J Mol Sci. 2021 Jun 3;22(11):6037. doi: 10.3390/ijms22116037 (PMC8199737; doi:10.3390/ijms22116037)
Supplement: Supplementary file 1 [file ijms-22-06037-s001.zip › ijms-1225011-supplementary.pdf]

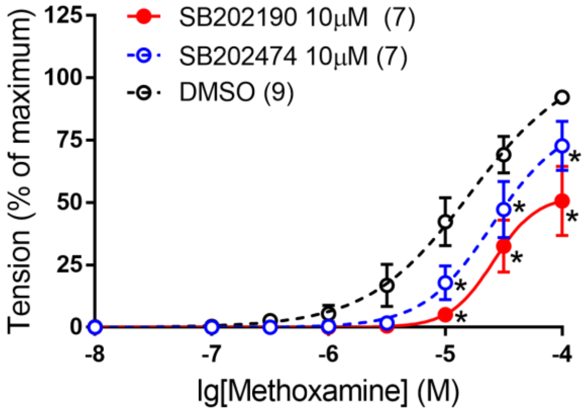

Figure S1. Concentration-response relationships to MX of endothelium-intact arteries from 1- to 2-week-old rats in the presence of the p38 MAPK inhibitor SB202190 (10 μM), or its inactive analogue SB202474 (10 μM) or their vehicle DMSO. * $p < 0.05$ vs. DMSO (Repeated Measures ANOVA).
